# Prediction of Antitubercular Peptides From Sequence Information Using Ensemble Classifier and Hybrid Features

**DOI:** 10.3389/fphar.2018.00954

**Published:** 2018-08-28

**Authors:** Salman Sadullah Usmani, Sherry Bhalla, Gajendra P. S. Raghava

**Affiliations:** ^1^Center for Computational Biology, Indraprastha Institute of Information Technology, New Delhi, India; ^2^Bioinformatics Centre, CSIR-Institute of Microbial Technology, Chandigarh, India

**Keywords:** tuberculosis, antitubercular peptides, machine learning, antimycobacterial therapy, *Mycobacterium*, ensemble classifier, drug discovery

## Abstract

Tuberculosis is one of the leading cause of death worldwide, particularly due to evolution of drug resistant strains. Antitubercular peptides may provide an alternate approach to combat antibiotic tolerance. Sequence analysis reveals that certain residues (e.g., Lysine, Arginine, Leucine, Tryptophan) are more prevalent in antitubercular peptides. This study describes the models developed for predicting antitubercular peptides by using sequence features of the peptides. We have developed support vector machine based models using different sequence features like amino acid composition, binary profile of terminus residues, dipeptide composition. Our ensemble classifiers that combines models based on amino acid composition and N5C5 binary pattern, achieves highest Acc of 73.20% with 0.80 AUROC on our main dataset. Similarly, the ensemble classifier achieved maximum Acc 75.62% with 0.83 AUROC on secondary dataset. Beside this, hybrid model achieves Acc of 75.87 and 78.54% with 0.83 and 0.86 AUROC on main and secondary dataset, respectively. In order to facilitate scientific community in designing of antitubercular peptides, we implement above models in a user friendly webserver (http://webs.iiitd.edu.in/raghava/antitbpred/).

## Introduction

Tuberculosis (TB) is one of the most ancient infectious disease of mankind caused by *Mycobacterium tuberculosis* (*M. tuberculosis*). DNA sequencing of a 17,870 ± 230 years old fossil of an extinct bison (Pleistocene bison), confirmed the existence of tuberculosis over thousands of years (Rothschild et al., 2001). ‘WHO Global Tuberculosis Report-2017' declared TB as one of the top 10 cause of death worldwide. In 2016, 1.7 million people died from TB and there were an estimated 10.4 million new (incident) TB cases worldwide among which 2.79 million were accounted for India. It is estimated that about 40% of the Indian population is infected with TB bacteria, the vast majority of whom have latent TB rather than TB disease (TB Statistics India | National, treatment outcome and state statistics)^1^. India, Indonesia, China, Philippines, Pakistan, Nigeria, and South Africa are accounted for 64% of the estimated new cases, making TB as major threat to the developing nations. The aerosolization release of viable airborne bacilli from the individuals with active tuberculosis, transmits it to the healthy individuals, with potential to further progress in disease (Churchyard et al., 2017). Therefore, an estimated one third population act as reservoir for TB (Teng et al., 2015).

Streptomycin was discovered as the first effective antibiotic against tuberculosis in 1944, but very soon the strains resistance to streptomycin was reported (Dickinson, 1947; Sandhu, 2011). From onwards, number of antibiotics such as isoniazid, rifampicin etc has been reported with significant initial success, but resistance is always an issue. In 1974, WHO has approved the use of BCG vaccine worldwide, to eradicate the TB, but its efficacy decreases with time (Kernodle, 2010) and found to be least effective in adults of tropical and subtropical region along with immune-compromised individuals (Andersen and Doherty, 2005). Currently, a combination of six first-line drugs is given for a very long duration, ~12 months (Wang et al., 2015). Failure of this treatment, persuade use of second-line drugs which are more toxic and less tolerable with severe side effects (van den Boogaard et al., 2009; Arbex et al., 2010). Evolution of multiple drug resistant (MDR), extremely drug resistant (XDR) and totally drug resistant (TDR) strain makes the scenario worst. Therefore, it's an urgent need to develop new anti-mycobacterial therapies. One of the possible alternative is peptide-based therapies. The most important aspect of peptides are their ability to bind range of biological targets, including *in vivo* molecular entities, leading to high potency with lower toxicity, making them better medicinal candidate than small molecules (Usmani et al., 2017). Beside this, low immunogenicity of anti-mycobacterial peptides make them a possible alternate or supplement for conventional TB drugs (AlMatar et al., 2018). These antimycobacterial peptides have selective affinity to cell envelope as well as targeted immune response against *Mycobacterium* (Teng et al., 2015).

Intensified interest in peptide-based therapies forces, both researchers and pharmaceutical industries, to hasten the designing of newer peptides. Therefore, to assist them, a number of *in silico* tools to predict and design various kind of therapeutic peptide such as cell-penetrating, tumor-hoping, anti-microbial, anti-bacterial, anti-fungal, vaccine, immunotherapy, etc. has been developed in recent years (Lata et al., 2007; Sharma et al., 2013; Dhanda et al., 2017; Agrawal et al., 2018; Kumar et al., 2018; Usmani et al., 2018a). *Mycobacterium*, neither Gram-positive nor Gram-negative, has unusual waxy coating (primarily of mycolic acid) on the cell surface, being dissimilar to other bacteria (Bhat et al., 2017; Squeglia et al., 2018; Velayati et al., 2018). The distinguish characteristic of *Mycobacterium* make them inappropriate for universal anti-bacterial peptide prediction methods. Consequently, in the current study, an attempt has been made to develop models using machine learning techniques for discriminating anti-tubercular (or anti-mycobacterial peptides) with other anti-bacterial peptides (ABP) as well non-antibacterial peptides (non-ABP).

## Materials and methods

### Dataset preparation

**Figure 1:**
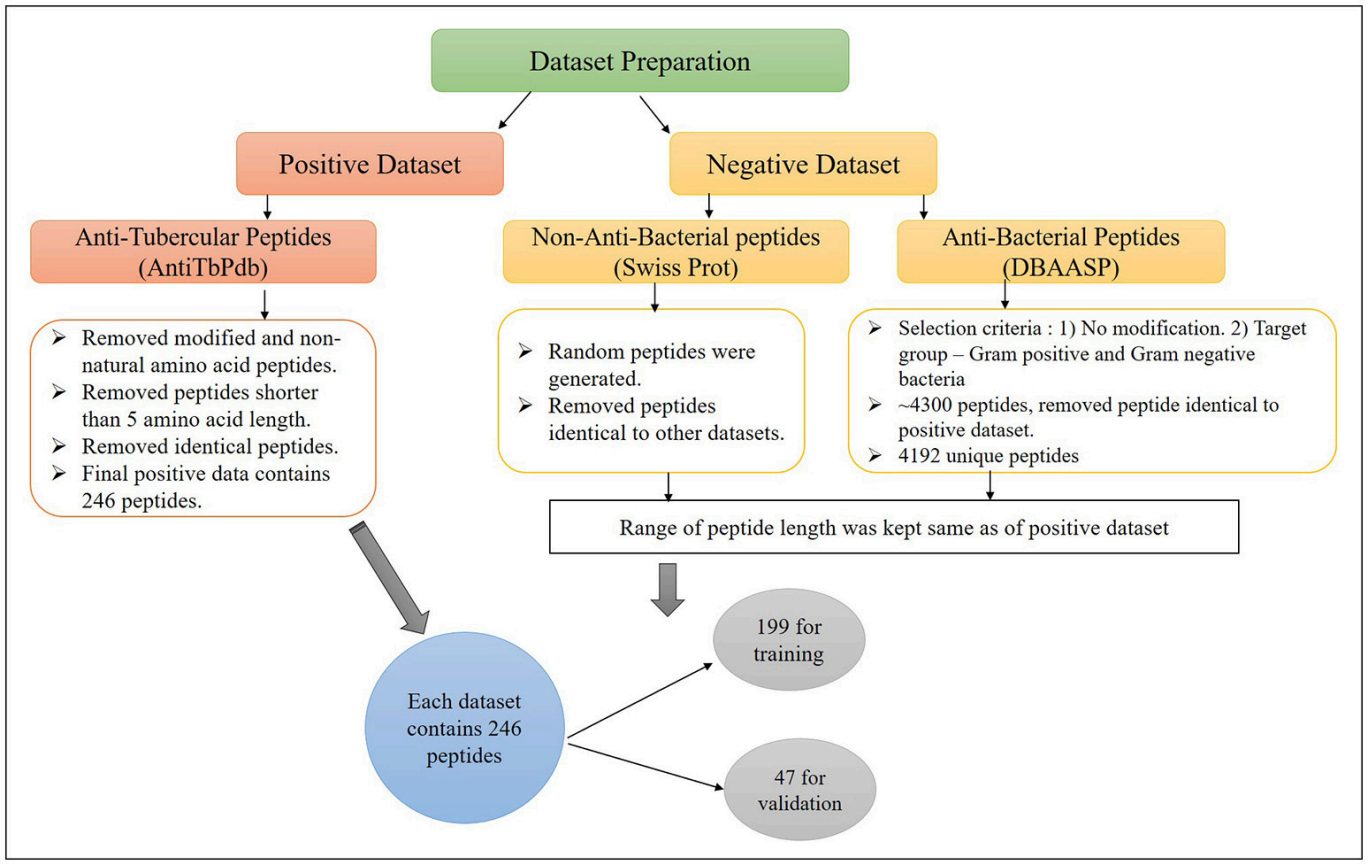
The construction of positive and negative dataset to develop machine learning models for prediction of Anti-tubercular peptides.

The major challenge of developing bioinformatic tool is to get the adequate amount of accurate experimental data. In this study, we have extracted anti-tubercular peptides (AntiTbP), from AntiTbPdb; a manually curated database of experimentally verified AntiTbP (Usmani et al., 2018b). Most of the curated peptides, in AntiTbPdb contains non-natural modifications, but we have taken peptides with natural amino acid only. After removing the identical peptides, final positive data consist of 246 unique peptides, varies in length of 5–61, effective against *Mycobacterium* (Figure 1). For negative dataset, we have prepared two separate datasets; (i) AntiTb_MD, which is prepared from DBAASP; an antimicrobial peptide (AMP) database (Gogoladze et al., 2014; Pirtskhalava et al., 2016) and (ii) AntiTb_RD, which is prepared from Swiss-Prot (Bairoch and Apweiler, 2000). From DBAASP, we have selected peptides containing natural amino acids without any modifications and are active against Gram positive and Gram negative bacteria. After removing the redundancy as well as AntiTbP (identical to positive dataset) 4192 unique peptides were left. From this, we have generated one of our negative dataset, containing 246 anti-bacterial peptides only. Beside this, 246 random peptides were generated from Swiss-Prot. While generating the random peptides; peptides identical to AntiTbP and ABP were removed, making it non-ABP dataset. The range of peptide length was kept same in all three datasets. By generating different bins (5–14, 15–24 etc.), we ensured that almost same number of equal length of peptides, must be present in bins of all the datasets. All these datasets were randomly divided into two parts, in such a manner, that almost all length range must be included in both; (i) training dataset, which contain 80% of data (199 sequences) and (ii) validation dataset, comprising of 20% of data (47 sequences) (Supplementary Table S1).

### Internal and external validation

For internal validation, we used standard five-fold cross validation technique, in which whole dataset is divided into five equal parts. The four dataset are used for training, whereas remaining one is used for testing. The process continues till each set is used for testing and the final result is calculated by averaging the performance of all the five sets (Nagpal et al., 2017). The external validation of any prediction method plays a very significant role in its evaluation. We have used 20% of our data (i.e., validation dataset) for external validation. Validation dataset is defined as sample of data, held back from training our model. In machine learning, it is used to give an estimate of model performance while tuning model's parameters. We too have evaluated the performance of all the models on validation datasets.

### Sequence logo

The sequence logos were generated using online Seq2Logo webserver (Nagpal et al., 2017). These are the graphical representation of sequences, which gives position specific frequency of amino acids in the multiple peptide sequences. There is a stack of symbols representing the amino acid at each positions. Large symbols represent frequently observed amino acids, big stacks represents conserved positions and small stacks represents variable positions.

### Computation of features for prediction

Peptide features such as amino acid composition (AAC), dipeptide composition (DPC), split composition and binary profiles were used to develop prediction models.

#### Amino acid composition

AAC has been successfully applied in various sequence-based classification algorithms (Soga et al., 2007; Gupta et al., 2013; Kumar et al., 2017; Manavalan et al., 2017, 2018a,c). AAC summarizes the peptide information in a vector of 20 dimensions. It is the fraction of each type of amino acid with in a peptide and is calculated by the following equation;

$$\begin{array}{l} AAC\left(a\right)=\frac{Ra}{N}x100 \end{array}$$

Where, AAC (a) is the percent composition of amino acid (a); R_a_ is the numbers of residues of type a, and N represents the total number of peptide's residues.

#### Dipeptide composition

It gives the composition of pair of residues (e.g., Gly-Gly, Gly-Leu, etc.) present in peptide. DPC transform the variable length of peptide to a fixed pattern of 400 vectors and summarizes fraction of amino acids as well as their local order. It was calculated by using the following equation;

$$\begin{array}{l} Fraction\;of\;Dipeptide\;\left(a\right)\;=\\ \frac{Total\;number\;of\;Dipeptide\left(a\right)}{Total\;number\;ofall\;possible\;dipeptides}\times 100 \end{array}$$

Where dipeptide (a) is one out of 400 dipeptides.

#### Terminus composition

Five amino acids from each N-terminal and C-terminal end of peptides were considered to calculate the N5 and C5-amino acid composition respectively. Beside this, we have joined the terminal residues as N5C5 and its AAC is also considered as feature to develop prediction model.

#### Binary profile of patterns

Previously, several studies shows the importance of binary profiling while developing prediction methods (Agrawal et al., 2018). The binary profile encapsulates information of both composition as well as order of amino acids in peptides. Binary profiles were generated for each peptide, where each amino acid is represented by a vector of dimensions of 20 (e.g., Ala by 1,0,0,0,0,0,0,0,0,0,0,0,0,0,0,0,0,0,0,0). A pattern of window length W was represented by a vector of dimensions 20 × W. Our dataset consist of a varied length of peptides, ranging from 5 to 61, therefore a fixed length of binary vector is not possible. To overcome this, we have extracted 5 amino acid from terminus of each peptides to cover all the peptides. Beside these N5 and C5 sequences, a concatenated derived sequence (N5C5) were also used to generate the binary profile.

### Machine learning techniques

We used SVMlight package, consisting of various kernels, to develop the Support vector machine (SVM) based prediction models (Joachims and Thorsten, 2002). SVM requires fixed length of input features from training data. The maximum information about peptides of variable length were converted into fixed vector of same dimensions (AAC, DPC, Binary profiling) were used as input features. We have augmented range of parameters to get the best performance on training dataset. Subsequently, best learned model was used for validation. In addition to SVM, different classifiers (e.g., Random Forest (RF), SMO, J48, and Naïve Bayes) unified in WEKA suite were also used to develop prediction models. Weka package has been used to implement these classifiers (Witten et al., 2016). All these machine learning methods have been successfully applied in many bioinformatics studies (Manavalan et al., 2014, 2018b; Chen et al., 2017; Lin et al., 2017; Manavalan and Lee, 2017; Zhao et al., 2017).

### Performance evaluation parameters

Both type of threshold dependent and independent parameters were used to evaluate the performance of each model developed in the study.

#### Threshold dependent parameters

Sensitivity (Sen), Specificity (Spc), Accuracy (Acc), and Matthews's correlation coefficient (MCC) are the threshold dependent parameters. “Sen” is defined as true positive rate whereas true negative rate is defined by “Spc.” “Acc” is ability to differentiate true positive and true negative while MCC is a correlation coefficient between observed and predicted values. These can be calculated using the following equations.

$$\begin{array}{lll} \text{Sen} & = & \frac{TP}{PS}\times 100\\ \text{Spc} & = & \frac{TN}{NS}\times 100\\ \text{Acc} & = & \frac{TP+TN}{PS+NS}\times 100\\ \text{MCC} & = & \frac{1-\left(\frac{FN}{PS}\times \frac{FP}{NS}\right)}{\sqrt{\left(1+\frac{FP-FN}{PS}\right)\times \left(1+\frac{FN-FP}{NS}\right)}} \end{array}$$

Where TP represents correctly predicted positive, TN represents the negative examples, PS represents total sequences in positive set, NS represents total sequences in negative set, FP represents actual negative examples which have been wrongly predicted as positive, and FN represents wrongly predicted positive examples. This is a well-established method of measuring performance and has been used earlier in many studies (Kumar et al., 2018).

#### Threshold independent parameters

Area under Receiver Operating Characteristics (AUROC) value; a threshold independent measure, is calculated between false positive and false negative rates (Kumar et al., 2018).

### Statistical analysis

Wilcoxon signed-rank test was utilized to assess the significance differences between sets of different AUROC values.

## Results

### Peptide compositional analysis

**Figure 2:**
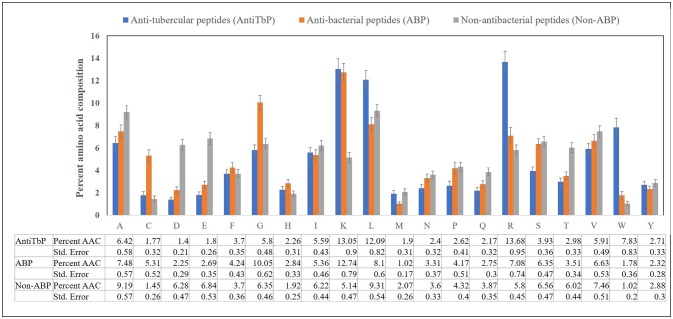
Comparison of percent amino acid composition of anti-tubercular, antibacterial, and non-antibacterial peptides.

Compositional analysis of peptides is very significant in identifying the nature of peptide. Compositional analysis reveals dominance of lysine (K), arginine (R), leucine (L), and tryptophan (W) amino acid in AntiTbP. Similarly, ABP also contains cysteine (C), glycine (G), lysine (K), and arginine (R) in higher propensity than non-ABP (Figure 2). The percentage of L and R are high in both ABP and AntiTbP, but the percentage of C, G, L, and W might be the reason behind the difference in nature.

### Positional residue preference analysis

**Figure 3:**
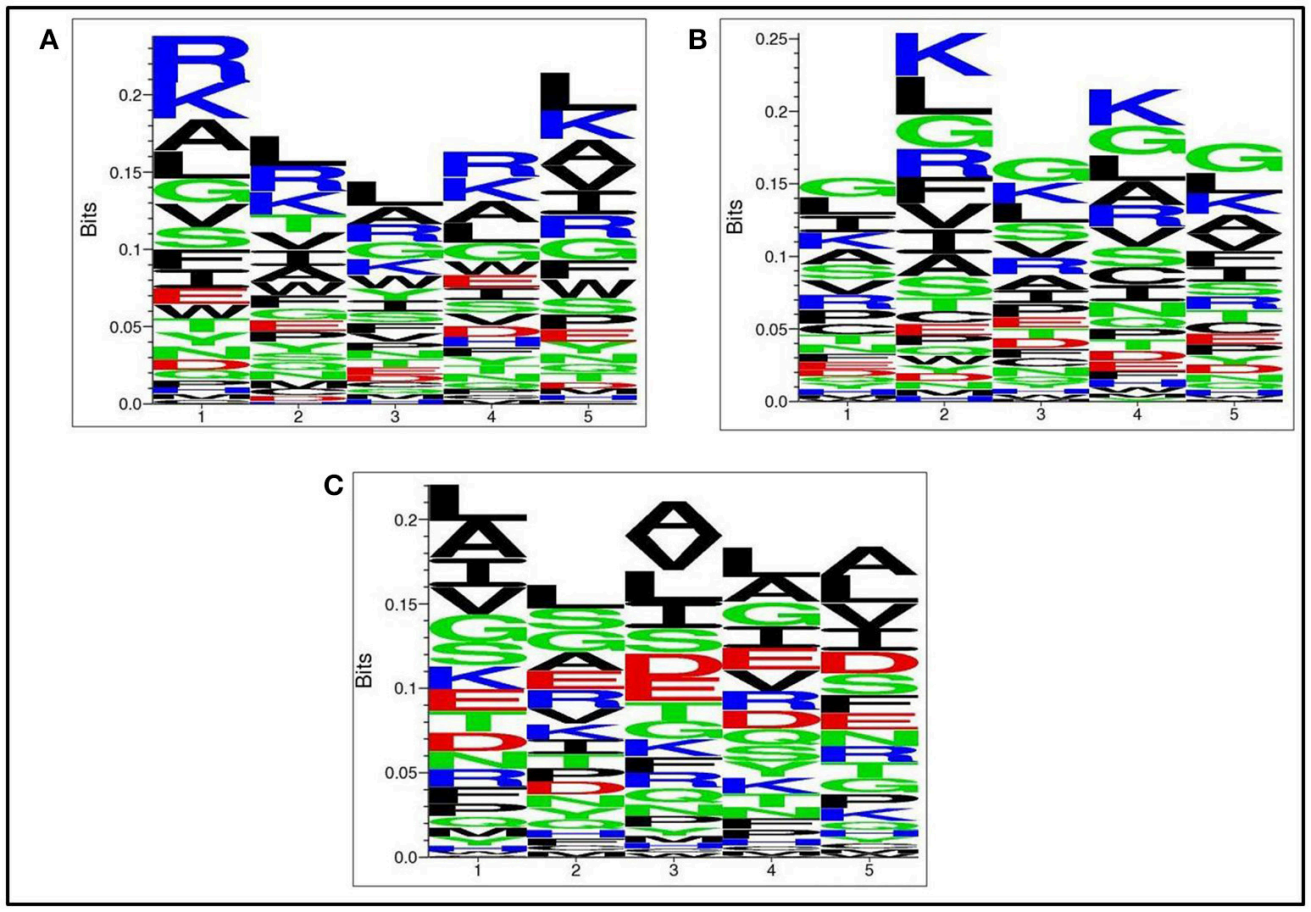
Comparison of residue preference at N-terminal of **(A)** Anti-tubercular, **(B)** Anti-bacterial, and **(C)** Non-antibacterial peptide.

**Figure 4:**
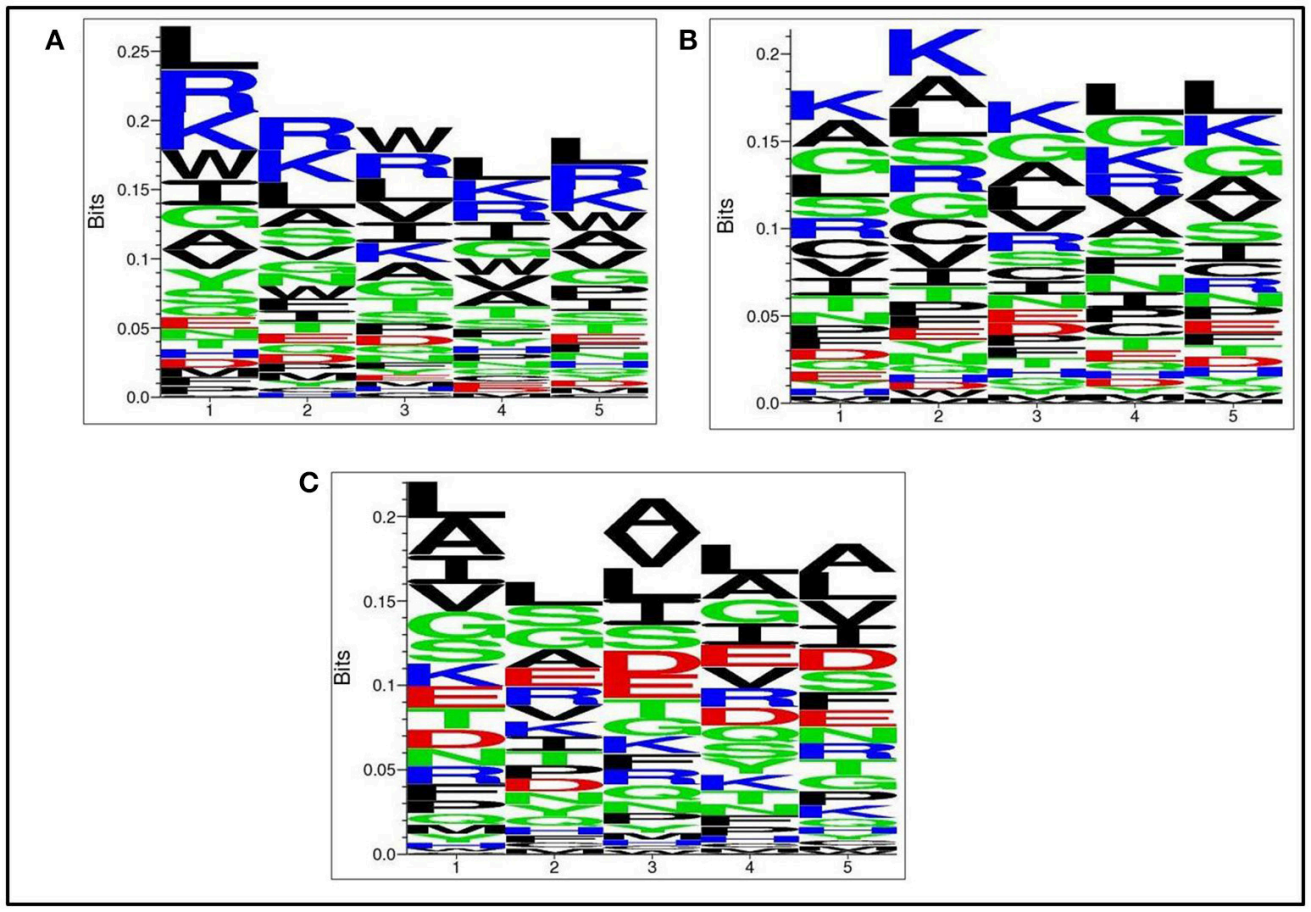
Comparison of residue preference at C terminal of **(A)** Anti-tubercular, **(B)** Anti-bacterial, and **(C)** Non-antibacterial peptide.

Next, we analysed, which types of residues are preferred at specific positions in AntiTbP as compared to other ABP. Frequency of occurrence of amino acids at N5 and C5 terminal end was examined to comprehend the difference (Figures 3, 4). In case of AntiTbP, R is the most preferred amino acid at position 1 and 4, whereas L is preferred at position 2, 3, and 5 at the N-terminal end. K is preferred at 2nd and 4th position while G is found frequently 1st, 3rd, and 5th position at N-terminal of ABP. Similarly, at C terminus of AntiTbP, L is preferred at 1st, 4th, and 5th position while at 2nd and 3rd position, R and W are preferred respectively. In case of ABP, K is preferred at 1st, 2nd, and 3rd position while at 4th and 5th position, L is the most preferred amino acid.

### Machine learning based prediction models

Various machine-learning approaches like SVM, RF, Naive Bayes, J48, and SMO have been used for developing prediction models. These models employ different features to discriminate AntiTbP with ABP as well as non-ABP. The results are explained in details in the following sections.

#### Models for discriminating AntiTbP from Non-ABP

As illustrated in material and method section, we have used random peptides (non-ABP) as negative dataset (AntiTb_RD) to differentiate between AntiTbP and non-ABP. Different features like AAC, DPC, and terminal residue compositions and binary pattern has been used as input feature to develop prediction methods.

In case of AAC based models, SVM technique gives 81.41% Acc, 0.63 MCC with 0.85 AUROC on training dataset whereas 79.79% Acc, 0.62 MCC with 0.88 AUROC on validation dataset. RF gives 74.87% Acc with 0.85 AUROC on training dataset while on validation Acc and AUROC reaches to 89.36%, and 0.94, respectively (Table 1). Similarly, Acc of 81.91, 78.72, and 81.91%, whereas 0.82, 0.87, and 0.88 AUROC are achieved on validation dataset by SMO, Naïve-Bayes, and J48, respectively. DPC as input feature gives 81.91% Acc on validation dataset by both SVM and RF method. SMO, Naïve-Bayes and J48 gives 79.79, 79.79, and 77.66% Acc with 0.80, 0.80, and 0.81 AUROC values (Supplementary Table S2),

**Table 1:** The performance of different machine learning techniques based models on AntiTb_RD dataset developed using AAC of peptides.

**Technique**		**Sen**	**Spc**	**Acc**	**MCC**	**AUROC**
SVM	Train	78.39	84.42	81.41	0.63	0.85
	Valid	65.96	93.62	79.79	0.62	0.88
RF	Train	74.87	74.87	74.87	0.50	0.85
	Valid	87.23	91.49	89.36	0.79	0.94
SMO	Train	75.88	80.40	78.14	0.56	0.78
	Valid	80.85	82.98	81.91	0.64	0.82
NB	Train	67.84	90.45	79.15	0.60	0.84
	Valid	63.83	93.62	78.72	0.60	0.87
J48	Train	67.84	75.38	71.61	0.43	0.75
	Valid	82.98	80.85	81.91	0.64	0.88

We have also used 5 amino acid from both N and C terminal of the peptide as input features. In case of N5 AAC, 0.82, 0.83, 0.72, 0.84, and 0.85 AUROC is achieved by SVM, RF, SMO, Naïve- Bayes, and J48 respectively on validation dataset (Supplementary Table S3). Likewise, C5 AAC gives 0.81, 0.76, 0.64, 0.81, and 0.73 AUROC values on validation dataset by SVM, RF, SMO, Naïve- Bayes, and J48 respectively (Supplementary Table S4). In addition to this, the catenated N5C5 gives, 0.84, 0.90, 0.80, 0.89, and 0.82 AUROC values with 80.85, 79.79, 79.79, 84.04, and 74.47% Acc by SVM, RF, SMO, Naïve- Bayes, and J48 respectively (Supplementary Table S5).

With the aim of considering amino acid order in peptide, binary patterns of N5 and C5 terminal end were generated and used as input features by different machine learning techniques. The N5 terminal binary pattern gives AUROC of 0.81, 0.81, 0.72, 0.84, and 0.70 by SVM, RF, SMO, Naïve- Bayes, and J48 respectively on validation dataset (Supplementary Table S6). Similarly, on validation dataset, C5 terminal binary pattern gives 0.86, 0.78, 0.76, 0.82, and 0.71 AUROC values by SVM, RF, SMO, Naïve- Bayes, and J48 respectively (Supplementary Table S7). The catenated N5C5 binary pattern consider the order of amino acid at both end of peptides, therefore also implemented as input features in our study (Table 2). On validation dataset, It gives 79.79, 80.85, 82.98, 82.98. and 67.02% Acc with 0.88, 0.89, 0.83, 0.91. and 0.68 AUROC values by SVM, RF, SMO, Naïve- Bayes. and J48 respectively.

**Table 2:** The performance of different machine learning techniques based models on AntiTb_RD dataset developed using binary pattern of peptide segments obtained from N and C terminals.

**Technique**		**Sen**	**Spc**	**Acc**	**MCC**	**AUROC**
SVM	Train	72.86	81.91	77.39	0.55	0.82
	Valid	70.21	89.36	79.79	0.61	0.88
RF	Train	73.87	78.39	76.13	0.52	0.82
	Valid	72.34	89.36	80.85	0.63	0.89
SMO	Train	70.85	80.40	75.63	0.51	0.76
	Valid	74.47	91.49	82.98	0.67	0.83
NB	Train	62.81	89.45	76.13	0.54	0.82
	Valid	68.09	97.87	82.98	0.69	0.91
J48	Train	72.36	66.33	69.35	0.39	0.68
	Valid	70.21	63.83	67.02	0.34	0.68

To overcome any false prediction, we have also implemented support vector machine based ensemble approach. As mentioned earlier, Non-ABP were generated from, Swiss-Prot, therefore, to maintain the sequential diversity in negative dataset, we have generated five different negative datasets and used in five different runs. As we have achieved significant performance by using AAC and N5C5 binary patterns, the SVM scores of both these models were average to get final model. The process was accomplished on all five different datasets and the average Acc achieved is 77.47% with 0.85 AUROC and 0.56 MCC on training dataset while 75.62% Acc, 0.52 MCC and 0.83 AUROC is achieved on validation dataset (Table 3).

**Table 3:** The SVM based ensemble of AAC and N5C5 binary pattern on AntiTb_RD on five different training and validation datasets along with average results.

	**Training**					**Validation**				
	**Sen**	**Spc**	**Acc**	**MCC**	**AUROC**	**Sen**	**Spc**	**Acc**	**MCC**	**AUROC**
Run 1	69.19	88.38	78.79	0.59	0.86	62.50	79.17	70.83	0.42	0.78
Run 2	69.70	86.87	78.28	0.57	0.87	70.83	79.17	75.00	0.50	0.85
Run 3	69.19	87.37	78.28	0.58	0.86	72.92	83.33	78.12	0.57	0.81
Run 4	64.65	80.30	72.47	0.46	0.82	62.50	83.33	72.92	0.47	0.82
Run 5	71.21	87.88	79.55	0.60	0.86	77.08	85.42	81.25	0.63	0.89
Average	68.79	86.16	77.47	0.56	0.85	69.17	82.08	75.62	0.52	0.83

In addition to ensemble model, we have also constructed an hybrid model by combining AAC and N5C5 binary pattern features. This model is generated to compare the performance with ensemble approach. The same dataset used in each run of ensemble approach is used here, and the average performance is comparable to ensemble classifier (Table 4). The average Acc achieved is 81.66% with 0.87 AUROC on training dataset while 78.54% with 0.86 AUROC on validation dataset.

**Table 4:** The SVM based on hybrid features of AAC and N5C5 binary pattern on AntiTb_RD on five different training and validation datasets along with average results.

	**Training**					**Validation**				
	**Sen**	**Spc**	**Acc**	**MCC**	**AUROC**	**Sen**	**Spc**	**Acc**	**MCC**	**AUROC**
Run 1	78.28	83.84	81.06	0.62	0.88	70.83	87.50	79.17	0.59	0.85
Run 2	78.28	86.36	82.32	0.65	0.88	70.83	79.17	75.0	0.50	0.82
Run 3	80.81	83.84	82.32	0.65	0.87	77.08	81.25	79.17	0.58	0.86
Run 4	74.24	82.32	78.28	0.57	0.85	70.83	81.25	76.04	0.52	0.84
Run 5	81.82	86.87	84.34	0.69	0.88	77.08	89.58	83.33	0.67	0.92
Average	78.68	84.64	81.66	0.64	0.87	73.33	83.75	78.54	0.57	0.86

#### Models for discriminating antiTbP from ABP

The main aim of the study is to differentiate AntiTbP from general ABP. To accomplish this, various machine learning approaches on range of input features, as AAC, DPC, terminal amino acid composition and binary patterns have been implemented. AAC as input features gives 74.37, 76.63, 74.37, 68.09, and 73.87% Acc with 0.78, 0.84, 0.74, 0.74, and 0.76 AUROC on independent dataset by SVM, RF, SMO, Naïve- Bayes, and J48 respectively. While on validation dataset, 80.21, 72.92, 85.42, 67.71, and 70.83% Acc with 0.86, 0.78, 0.85, 0.73, and 0.74 MCC is achieved by SVM, RF, SMO, Naïve- Bayes, and J48 respectively (Table 5).

**Table 5:** The performance of different machine learning techniques based models on AntiTb_MD dataset developed using AAC of peptides.

**Technique**		**Sen**	**Spc**	**Acc**	**MCC**	**AUROC**
SVM	Train	78.39	70.35	74.37	0.49	0.78
	Valid	83.33	77.08	80.21	0.61	0.86
RF	Train	75.88	77.39	76.63	0.53	0.84
	Valid	72.92	72.92	72.92	0.46	0.78
SMO	Train	74.37	74.37	74.37	0.49	0.74
	Valid	83.33	87.50	85.42	0.71	0.85
NB	Train	58.79	77.39	68.09	0.37	0.74
	Valid	50.00	85.42	67.71	0.38	0.73
J48	Train	74.37	73.37	73.87	0.48	0.76
	Valid	70.83	70.83	70.83	0.42	0.74

DPC is also used as input features to develop models based on SVM, RF, SMO, Naïve- Bayes, and J48 techniques and gives 0.82, 0.76, 0.72, 0.66, and 0.69 AUROC respectively on validation dataset (Supplementary Table S8). When AAC of N5 terminus of peptide is used as input feature, 0.79, 0.78, 0.73, 0.69, and 0.69 AUROC is achieved on training dataset, while 0.67, 0.65, 0.63, 0.71, and 0.53 AUROC on validation dataset by SVM, RF, SMO, Naïve- Bayes, and J48 respectively (Supplementary Table S9). Similarly, C5 terminal AAC gives 0.76, 0.74, 0.70, 0.71, and 0.65 AUROC by SVM, RF, SMO, Naïve- Bayes, and J48 respectively on validation dataset (Supplementary Table S10), Beside this, N5C5 catenated features gives 0.79, 0.77, 0.73, 0.73, and 0.64 AUROC values on validation by SVM, RF, SMO, Naïve- Bayes, and J48 respectively (Supplementary Table S11).

The binary patterns of N5 terminal gives 0.73, 0.67, 0.70, 0.71, and 0.58 AUROC values while binary pattern of C5 terminal gives 0.72, 0.74, 0.73, 0.69, and 0.66 AUROC with the help of SVM, RF, SMO, Naïve- Bayes, and J48 respectively on validation (Supplementary Tables S12, 13). To encapsulate the maximum information about order of amino acid, the catenated N5C5 binary patterns were also used to develop model. In case of SVM, 73.37% Acc with 0.81 AUROC and 73.96% Acc with 0.80 AUROC is obtained on training and validation dataset respectively. RF gives 80.00% Sen and both Spc and Acc as 72.36% with 0.78 AUROC on training dataset, whereas on validation dataset 71.88% Acc with 0.75 AUROC is obtained. Similarly on validation, SMO, Naïve Bayes and J48 gives 0.77, 0.73 and 0.72 AUROC respectively (Table 6).

**Table 6:** The performance of different machine learning techniques based models on AntiTb_MD dataset developed using binary pattern of peptide segments obtained from N and C terminals.

**Technique**		**Sensitivity**	**Specificity**	**Acc**	**MCC**	**AUROC**
SVM	Train	69.85	76.88	73.37	0.47	0.81
	Valid	75.00	72.92	73.96	0.48	0.80
RF	Train	80.00	72.36	72.36	0.45	0.78
	Valid	77.08	66.67	71.88	0.44	0.75
SMO	Train	67.34	72.36	69.85	0.40	0.70
	Valid	72.92	81.25	77.08	0.54	0.77
NB	Train	56.28	78.89	67.59	0.36	0.73
	Valid	53.27	84.42	68.84	0.40	0.73
J48	Train	66.33	63.82	65.08	0.30	0.68
	Valid	68.75	70.83	69.79	0.40	0.72

In case of SVM based ensemble approach, AAC with N5C5 binary patterns were used as input features to classify AntiTbP from ABP. In this case, the negative dataset is reshuffled in five different runs, to check the impact of reshuffling of folds on the performance of model. The average Sen, Spc, Acc, and AUROC were 80.20, 72.89, 76.56% and 0.83 respectively were achieved on five different training datasets. In case of validation, 78.75% sensitivity, 67.76% specificity, 73.20% Acc with 0.80 AUROC were obtained (Table 7).

**Table 7:** The SVM based ensemble of AAC and N5C5 binary pattern on AntiTb_MD on five different training and validation datasets along with average results.

	**Training**					**Validation**				
	**Sen**	**Spc**	**Acc**	**MCC**	**AUROC**	**Sen**	**Spc**	**Acc**	**MCC**	**AUROC**
Run 1	82.83	76.14	79.49	0.59	0.85	72.92	67.35	70.10	0.40	0.78
Run 2	80.30	73.60	76.96	0.54	0.85	77.08	77.55	77.32	0.55	0.82
Run 3	78.79	73.60	76.20	0.52	0.84	85.42	51.02	68.04	0.39	0.72
Run 4	80.81	70.56	75.70	0.52	0.83	75.00	73.47	74.23	0.48	0.82
Run 5	78.28	70.56	74.43	0.49	0.81	83.33	69.39	76.29	0.53	0.84
Average	80.20	72.89	76.56	0.53	0.83	78.75	67.76	73.20	0.47	0.80

Beside this, a hybrid model combining AAC and N5C5 binary pattern features were also constructed and the same dataset used in each run of ensemble approach is used here, and the average performance is comparable to ensemble classifier (Table 8). The average Acc achieved is 77.48% with 0.82 AUROC on training dataset while 75.87% with 0.83 AUROC on validation dataset.

**Table 8:** The SVM based on hybrid features of AAC and N5C5 binary pattern on AntiTb_MD on five different training and validation datasets along with average results.

	**Training**					**Validation**				
	**Sen**	**Spc**	**Acc**	**MCC**	**AUROC**	**Sen**	**Spc**	**Acc**	**MCC**	**AUROC**
Run 1	79.29	73.68	78.99	0.58	0.85	70.83	71.43	71.13	0.42	0.81
Run 2	77.78	79.70	78.17	0.57	0.82	60.42	91.84	76.29	0.55	0.82
Run 3	75.76	78.17	76.96	0.54	0.83	85.42	61.22	73.20	0.48	0.80
Run 4	75.76	77.16	76.46	0.53	0.81	72.92	83.67	78.35	0.57	0.85
Run 5	74.24	77.66	75.95	0.52	0.79	85.42	75.51	80.41	0.61	0.88
Average	76.76	77.27	77.48	0.55	0.82	75.02	76.73	75.87	0.52	0.83

### Implementation of webserver

One of the major goals of the study is to provide service to the scientific community. Thus, we developed a user-friendly webserver (http://webs.iiitd.edu.in/raghava/antitbpred/) which will assist to know, whether a peptide has antitubercular activity. In addition to this, analogs of peptide can also be generated, with the possibility of being it as an AntiTbP or not, based on prediction score. Possibility of antitubercular peptide segments in a protein sequence can also be checked by using our webserver. We believe that, this webserver will be very useful to design newer AntiTbP as well as to know whether a known ABP can also have bactericidal activity against *Mycobacterium*.

## Discussion

Emergence of drug resistance, provoke the requirement of developing newer therapeutic strategies to combat tuberculosis. Last decade witnessed the advancement of several promising therapeutic entities. Antitubercular peptides emerged as promising anti-TB drugs, due to their selective affinity toward cell envelope and low immunogenicity and diverse mode of action (Teng et al., 2015). Beside, trans-membrane pore formation which is the common bactericidal mechanism, most of the AntiTbP tend to have intracellular targets such as both ecumicin and lassomycin act on ClpC1 ATPase complex (Gavrish et al., 2014; Gao et al., 2015). Most of the current AntiTbP are derived from bacterial extraction, mycobacteriophages or host immune cells; which is a tedious and costly process.

Peptidoglycan is the major component of *Mycobacterium* cell wall. A branched polysaccharide; named as Arabinogalactan, connects the peptidoglycan with the outer layer of mycolic acid. Some unique glycosyltransferases are involved in the cell wall assembly (Bhat et al., 2017). The unique structure of the cell wall plays an important role in the survival of *Mycobacterium*, while it enters into non-replicative growth throughout dormancy (Alderwick et al., 2015). This hydrophobic, waxy and thicker cell wall distinguish the *Mycobacterium* with other bacteria. Therefore, we believe that universal *in silico* tools, which were developed to predict AMP or ABP needs to be scrutinized thoroughly. To verify our concern, we have predicted the activity of experimentally validated AntiTbP by general prediction method incorporated in DBAASP as well as more improved method, iAMPpred; a recently developed tool to predict antimicrobial peptides (Meher et al., 2017). iAMPpred predicts 170 peptide as antibacterial whereas only 116 out of 246 experimentally validated AntiTbP are predicted as antimicrobial by DBAASP (Supplementary Table S14). These results clearly suggest that, it is need of an hour to develop, an exclusive method to design AntiTbP.

The amino acid compositional analysis reveals the preference of certain specific amino acid, such as K, L, R, and W in AntiTbP, whereas negatively charged D and E amino acid is less preferred. Analysis of positional preference of terminal residues, also emphasizes on the preference of R and L at N-terminal, and R, L, and W at C-terminal of the AntiTbP. The percentage of C, G, L, and W seems to be important while deciding the nature of peptides, to be ABP or AntiTbP. As the cell wall of *Mycobacterium* is highly negative charged, more cationic amino acids (K, L, and R) are required to perform the bactericidal activity. The difference in the composition of non-ABP, ABP, and AntiTbP, motivate us to develop methods to differentiate between AntiTbP with other peptides. In this study, we have used different input features such as AAC, DPC, terminal amino acid composition and binary pattern to develop several prediction models based on various machine learning techniques like SVM, RF, SMO, J48, and Naïve- Bayes. To avoid the false prediction of AAC based SVM model, (as two different peptide may have the same composition) and to consider the order of amino acids, we have implemented SVM based ensemble approach, in which five different training and validation sets have been used to construct set of SVM classifiers with the help of AAC and N5C5 binary patterns as input features, since they produced the best performance in real SVM. In case of antitubercular (positive) and antibacterial (negative) peptide- training dataset (AntiTb_MD), average Sen, Spc, Acc, MCC, and AUROC obtained are 80.20, 72.89, 76.56%, 0.53 and 0.83 respectively while on validation dataset, 78.75, 67.76, 73.20%, 0.47 and 0.80 corresponding values have been achieved. In the same way, 75.62% Acc with 0.83 AUROC has been achieved on validation dataset, comprising of antitubercular and non-antibacterial peptides (AntiTb_RD). There is a significant difference in performance of SVM based ensemble models and N5C5 binary pattern based model (*p* = 0.01), while the performance of hybrid model is almost same as ensemble (*p* = 0.52) (Table 9). Moreover, to assist the biologist, we have implemented our SVM based ensemble as well as hybrid models in a user-friendly web server to discriminate and design AntiTbP.

**Table 9:** *p*-values between AUROC of different methods obtained by implementing Wilcoxon rank sum test.

**S. No**		**Method 1**	**Method 2**	***p*-value**
1	AntiTb_RD dataset	Ensemble	SVM based on AAC	0.73
2		Ensemble	SVM based on N5C5 binary patterns	0.01
3		Ensemble	SVM based on hybrid features	0.1
4	AntiTb_MD dataset	Ensemble	SVM based on AAC	0.03
5		Ensemble	SVM based on N5C5 binary patterns	0.01
6		Ensemble	SVM based on hybrid features	0.52

The non-availability of negative data remains a major problem while developing prediction tools. We have tried to overcome this as much as possible while generating the negative data, with our assumptions, but availability of experimentally verified non-AntiTbP would have ensured more accurate performance. Similarly, the random peptide considered as non-ABP, might have bactericidal or even antitubercular activity, but this could only be confirmed after experimental verification. These are the flaws, which can only be overcome, when negative results (or negative peptide) will be reported as well as stored in a repository. The dataset is limited and consist of natural amino acids only. Inclusion of other novel natural as well modified AntiTbP will certainly provide a chance to improve the method.

In conclusion, the study bring about *in silico* models, to design AntiTbP (http://webs.iiitd.edu.in/raghava/antitbpred/). The models have advantages over general AMP and ABP prediction methods while predicting the bactericidal activity of peptides, specifically against *Mycobacterium*. The small dataset may be the limitation of the study, but we believe that with more characterization of AntiTbP, the field will grow significantly in the coming years.

## Author contributions

SU and SB generated the dataset, performed the experiment and data analysis. SU prepared figures and SB prepared tables. SU developed the web interface. SU and GR wrote the manuscript. GR conceived the idea and coordinated the project.

### Conflict of interest statement

The authors declare that the research was conducted in the absence of any commercial or financial relationships that could be construed as a potential conflict of interest.

## Footnotes

^1^TB Statistics India. *National, treatment outcome and state statistics*. (Accessed July 21, 2018). Available online at: https://www.tbfacts.org/tb-statistics-india/.
