# Supplementary material for: Prediction of Antitubercular Peptides From Sequence Information Using Ensemble Classifier and Hybrid Features
Source: Front Pharmacol. 2018 Aug 28;9:954. doi: 10.3389/fphar.2018.00954 (PMC6121089; doi:10.3389/fphar.2018.00954)
Supplement: Supplementary file 1 [file Table_1.DOCX]

Prediction of antitubercular peptides from sequence information using ensemble classifier and hybrid features

**Salman Sadullah Usmani ^1,2^, Sherry Bhalla ^1^ and Gajendra P.S. Raghava^*,1,2^**

1. Center for Computational Biology, Indraprastha Institute of Information Technology, Okhla, New Delhi-110020, India.
2. Bioinformatics Centre, CSIR-Institute of Microbial Technology, Sector-39A, Chandigarh- 160036, India.

**^*^ Corresponding author**

Professor, Center for Computational Biology, Indraprastha Institute of Information Technology, Okhla, New Delhi 110020, India.

India. Tel.: +91 011 26907444, Fax No. +91 11 26907405

**E-mail address:** [**raghava@iiitd.ac.in**](mailto:raghava@iiitd.ac.in)

Supplementary Table S1: Distribution of peptide length in all the dataset used throughout the study.

|  | Positive dataset | | Negative dataset (AntiTb_RD) | | Negative dataset (AntiTb_MD) | |
| --- | --- | --- | --- | --- | --- | --- |
|  | Training data | Validation data | Training data | Validation data | Training data | Validation data |
| Length range | Number of peptides | | | | | |
| 5-14 | 129 | 31 | 121 | 23 | 120 | 23 |
| 15-24 | 33 | 8 | 39 | 19 | 42 | 19 |
| 25-34 | 22 | 5 | 22 | 3 | 21 | 3 |
| 35-44 | 9 | 2 | 11 | 2 | 10 | 1 |
| 45-54 | 4 | 1 | 4 | 0 | 4 | 1 |
| 55-61 | 2 | 0 | 2 | 0 | 2 | 0 |
| Total | 199 | 47 | 199 | 47 | 199 | 47 |

Supplementary Table S2: The performance of different machine learning techniques based models on AntiTb_RD dataset developed using DPC of peptides.

| Technique | | Sen | Spc | Acc | MCC | AUROC |
| --- | --- | --- | --- | --- | --- | --- |
| SVM | Train | 78.89 | 86.43 | 82.66 | 0.66 | 0.87 |
|  | Valid | 74.47 | 89.36 | 81.91 | 0.65 | 0.88 |
| RF | Train | 81.41 | 85.93 | 83.67 | 0.67 | 0.88 |
|  | Valid | 80.85 | 82.98 | 81.91 | 0.64 | 0.91 |
| SMO | Train | 75.38 | 82.91 | 79.15 | 0.58 | 0.79 |
|  | Valid | 72.34 | 87.23 | 79.79 | 0.60 | 0.80 |
| NB | Train | 75.38 | 72.36 | 73.87 | 0.48 | 0.74 |
|  | Valid | 70.21 | 89.36 | 79.79 | 0.61 | 0.80 |
| J48 | Train | 73.87 | 70.85 | 72.36 | 0.45 | 0.72 |
|  | Valid | 80.85 | 74.47 | 77.66 | 0.55 | 0.81 |

Supplementary Table S3: The performance of different machine learning techniques based models on AntiTb_RD dataset developed using N5 composition of peptides.

| Technique | | Sen | Spc | Acc | MCC | AUROC |
| --- | --- | --- | --- | --- | --- | --- |
| SVM | Train | 72.86 | 75.88 | 74.37 | 0.49 | 0.81 |
|  | Valid | 74.47 | 70.21 | 72.34 | 0.45 | 0.82 |
| RF | Train | 74.37 | 76.88 | 75.63 | 0.51 | 0.81 |
|  | Valid | 82.98 | 68.09 | 75.53 | 0.52 | 0.83 |
| SMO | Train | 69.35 | 74.37 | 71.86 | 0.44 | 0.72 |
|  | Valid | 74.47 | 70.21 | 72.34 | 0.45 | 0.72 |
| NB | Train | 62.31 | 78.89 | 70.60 | 0.40 | 0.74 |
|  | Valid | 70.21 | 85.11 | 77.66 | 0.56 | 0.84 |
| J48 | Train | 72.36 | 67.84 | 70.10 | 0.40 | 0.69 |
|  | Valid | 82.98 | 74.47 | 78.72 | 0.58 | 0.85 |

Supplementary Table 4: The performance of different machine learning techniques based models on AntiTb_RD dataset developed using C5 composition of peptides.

| Technique | | Sen | Spc | Acc | MCC | AUROC |
| --- | --- | --- | --- | --- | --- | --- |
| SVM | Train | 77.89 | 83.92 | 80.90 | 0.62 | 0.86 |
|  | Valid | 68.09 | 89.36 | 78.72 | 0.59 | 0.81 |
| RF | Train | 78.89 | 81.41 | 80.15 | 0.60 | 0.87 |
|  | Valid | 72.34 | 74.47 | 73.40 | 0.47 | 0.76 |
| SMO | Train | 76.38 | 84.92 | 80.65 | 0.62 | 0.81 |
|  | Valid | 27.66 | 100.00 | 63.83 | 0.40 | 0.64 |
| NB | Train | 70.35 | 75.38 | 72.86 | 0.46 | 0.81 |
|  | Valid | 61.70 | 80.85 | 71.28 | 0.43 | 0.81 |
| J48 | Train | 74.37 | 79.40 | 76.88 | 0.54 | 0.77 |
|  | Valid | 63.83 | 76.60 | 70.21 | 0.41 | 0.73 |

Supplementary Table 5: The performance of different machine learning techniques based models on AntiTb_RD dataset developed using N5C5 composition of peptides.

| Technique | | Sen | Spc | Acc | MCC | AUROC |
| --- | --- | --- | --- | --- | --- | --- |
| SVM | Train | 76.38 | 82.41 | 79.40 | 0.59 | 0.85 |
|  | Valid | 72.34 | 89.36 | 80.85 | 0.63 | 0.84 |
| RF | Train | 77.89 | 81.41 | 79.65 | 0.59 | 0.87 |
|  | Valid | 80.85 | 78.72 | 79.79 | 0.60 | 0.90 |
| SMO | Train | 74.37 | 79.90 | 77.14 | 0.54 | 0.77 |
|  | Valid | 78.72 | 80.85 | 79.79 | 0.60 | 0.80 |
| NB | Train | 69.85 | 85.43 | 77.64 | 0.56 | 0.83 |
|  | Valid | 74.47 | 93.62 | 84.04 | 0.69 | 0.89 |
| J48 | Train | 79.90 | 77.89 | 78.89 | 0.58 | 0.82 |
|  | Valid | 72.34 | 76.60 | 74.47 | 0.49 | 0.82 |

Supplementary Table S6: The performance of different machine learning techniques based models on AntiTb_RD dataset developed using binary pattern of peptide segments obtained from N terminal.

| Technique | | Sen | Spc | Acc | MCC | AUROC |
| --- | --- | --- | --- | --- | --- | --- |
| SVM | Train | 67.34 | 74.87 | 71.11 | 0.42 | 0.75 |
|  | Valid | 68.09 | 82.98 | 75.53 | 0.52 | 0.81 |
| RF | Train | 64.32 | 66.33 | 65.33 | 0.31 | 0.71 |
|  | Valid | 78.72 | 63.83 | 71.28 | 0.43 | 0.81 |
| SMO | Train | 62.81 | 69.35 | 66.08 | 0.32 | 0.66 |
|  | Valid | 55.32 | 89.36 | 72.34 | 0.48 | 0.72 |
| NB | Train | 58.29 | 79.9 | 69.1 | 0.39 | 0.73 |
|  | Valid | 65.96 | 89.36 | 77.66 | 0.57 | 0.84 |
| J48 | Train | 60.3 | 60.3 | 60.3 | 0.21 | 0.59 |
|  | Valid | 74.47 | 63.83 | 69.15 | 0.39 | 0.70 |

Supplementary Table S7: The performance of different machine learning techniques based models on AntiTb_RD dataset developed using binary pattern of peptide segments obtained from C terminal.

| Technique | | Sen | Spc | Acc | MCC | AUROC |
| --- | --- | --- | --- | --- | --- | --- |
| SVM | Train | 69.35 | 78.39 | 73.87 | 0.48 | 0.80 |
|  | Valid | 70.21 | 85.11 | 77.66 | 0.56 | 0.86 |
| RF | Train | 78.89 | 71.36 | 75.13 | 0.50 | 0.82 |
|  | Valid | 70.21 | 63.83 | 67.02 | 0.34 | 0.78 |
| SMO | Train | 61.31 | 87.94 | 74.62 | 0.51 | 0.75 |
|  | Valid | 59.57 | 91.49 | 75.53 | 0.54 | 0.76 |
| NB | Train | 67.34 | 84.42 | 75.88 | 0.53 | 0.82 |
|  | Valid | 59.57 | 89.36 | 74.47 | 0.51 | 0.82 |
| J48 | Train | 77.89 | 61.31 | 69.60 | 0.40 | 0.69 |
|  | Valid | 72.34 | 68.09 | 70.21 | 0.40 | 0.71 |

Supplementary Table S8: The performance of different machine learning techniques based models on AntiTb_MD dataset developed using DPC of peptides.

| Technique | | Sen | Spc | Acc | MCC | AUROC |
| --- | --- | --- | --- | --- | --- | --- |
| SVM | Train | 78.39 | 76.38 | 77.39 | 0.55 | 0.84 |
|  | Valid | 79.17 | 70.83 | 75.00 | 0.50 | 0.82 |
| RF | Train | 77.39 | 81.91 | 79.65 | 0.59 | 0.87 |
|  | Valid | 81.25 | 66.67 | 73.96 | 0.48 | 0.76 |
| SMO | Train | 74.87 | 78.89 | 76.88 | 0.54 | 0.77 |
|  | Valid | 72.92 | 70.83 | 71.88 | 0.44 | 0.72 |
| NB | Train | 73.37 | 72.86 | 73.12 | 0.46 | 0.73 |
|  | Valid | 62.50 | 68.75 | 65.62 | 0.31 | 0.66 |
| J48 | Train | 72.86 | 72.86 | 72.86 | 0.46 | 0.73 |
|  | Valid | 79.17 | 62.50 | 70.83 | 0.42 | 0.69 |

Supplementary Table S9: The performance of different machine learning techniques based models on AntiTb_MD dataset developed using N5 composition of peptides.

| Technique | | Sen | Spc | Acc | MCC | AUROC |
| --- | --- | --- | --- | --- | --- | --- |
| SVM | Train | 70.47 | 79.19 | 74.87 | 0.50 | 0.79 |
|  | Valid | 51.06 | 74.47 | 62.77 | 0.26 | 0.67 |
| RF | Train | 72.02 | 69.04 | 70.51 | 0.41 | 0.78 |
|  | Valid | 68.09 | 55.32 | 61.70 | 0.24 | 0.65 |
| SMO | Train | 73.58 | 72.08 | 72.82 | 0.46 | 0.73 |
|  | Valid | 57.45 | 68.09 | 62.77 | 0.26 | 0.63 |
| NB | Train | 62.69 | 65.99 | 64.36 | 0.29 | 0.69 |
|  | Valid | 53.19 | 63.83 | 58.51 | 0.17 | 0.71 |
| J48 | Train | 70.98 | 67.01 | 68.97 | 0.38 | 0.69 |
|  | Valid | 48.94 | 59.57 | 54.26 | 0.09 | 0.53 |

Supplementary Table S10: The performance of different machine learning techniques based models on AntiTb_MD dataset developed using C5 composition of peptides.

| Technique | | Sen | Spc | Acc | MCC | AUROC |
| --- | --- | --- | --- | --- | --- | --- |
| SVM | Train | 70.92 | 79.70 | 75.32 | 0.51 | 0.82 |
|  | Valid | 66.67 | 72.92 | 69.79 | 0.40 | 0.76 |
| RF | Train | 71.43 | 72.08 | 71.76 | 0.44 | 0.80 |
|  | Valid | 72.92 | 66.67 | 69.79 | 0.40 | 0.74 |
| SMO | Train | 71.43 | 71.07 | 71.25 | 0.42 | 0.71 |
|  | Valid | 66.67 | 72.92 | 69.79 | 0.40 | 0.70 |
| NB | Train | 61.22 | 75.63 | 68.45 | 0.37 | 0.74 |
|  | Valid | 47.92 | 79.17 | 63.54 | 0.29 | 0.71 |
| J48 | Train | 66.84 | 69.04 | 67.94 | 0.36 | 0.69 |
|  | Valid | 81.25 | 56.25 | 68.75 | 0.39 | 0.65 |

Supplementary Table S11: The performance of different machine learning techniques based models on AntiTb_MD dataset developed using N5C5 composition of peptides.

| Technique | | Sen | Spc | Acc | MCC | AUROC |
| --- | --- | --- | --- | --- | --- | --- |
| SVM | Train | 68.84 | 75.88 | 72.36 | 0.45 | 0.77 |
|  | Valid | 72.92 | 72.92 | 72.92 | 0.46 | 0.79 |
| RF | Train | 72.36 | 72.36 | 72.36 | 0.45 | 0.80 |
|  | Valid | 72.92 | 68.75 | 70.83 | 0.42 | 0.77 |
| SMO | Train | 70.35 | 70.35 | 70.35 | 0.41 | 0.70 |
|  | Valid | 75.00 | 70.83 | 72.92 | 0.46 | 0.73 |
| NB | Train | 86.00 | 56.78 | 78.39 | 67.59 | 0.72 |
|  | Valid | 52.08 | 83.33 | 67.71 | 0.37 | 0.73 |
| J48 | Train | 66.33 | 69.35 | 67.84 | 0.36 | 0.69 |
|  | Valid | 66.67 | 56.25 | 61.46 | 0.23 | 0.64 |

Supplementary Table S12: The performance of different machine learning techniques based models on AntiTb_MD dataset developed using binary pattern of peptide segments obtained from N terminal.

| Technique | | Sen | Spc | Acc | MCC | AUROC |
| --- | --- | --- | --- | --- | --- | --- |
| SVM | Train | 67.88 | 71.07 | 69.49 | 0.39 | 0.73 |
|  | Valid | 65.96 | 72.34 | 69.15 | 0.38 | 0.73 |
| RF | Train | 66.84 | 69.04 | 67.95 | 0.36 | 0.75 |
|  | Valid | 68.09 | 55.32 | 61.70 | 0.24 | 0.67 |
| SMO | Train | 69.43 | 67.51 | 68.46 | 0.37 | 0.68 |
|  | Valid | 72.34 | 68.09 | 70.21 | 0.40 | 0.70 |
| NB | Train | 54.92 | 73.60 | 64.36 | 0.29 | 0.70 |
|  | Valid | 57.45 | 68.09 | 62.77 | 0.26 | 0.71 |
| J48 | Train | 57.51 | 65.48 | 61.54 | 0.23 | 0.63 |
|  | Valid | 55.32 | 70.21 | 62.77 | 0.26 | 0.58 |

Supplementary Table S13: The performance of different machine learning techniques based models on AntiTb_MD dataset developed using binary pattern of peptide segments obtained from C terminal.

| Technique | | Sen | Spc | Acc | MCC | AUROC |
| --- | --- | --- | --- | --- | --- | --- |
| SVM | Train | 70.92 | 70.05 | 70.48 | 0.41 | 0.78 |
|  | Valid | 66.67 | 77.08 | 71.88 | 0.44 | 0.72 |
| RF | Train | 71.43 | 65.99 | 68.70 | 0.37 | 0.76 |
|  | Valid | 75.00 | 64.58 | 69.79 | 0.40 | 0.74 |
| SMO | Train | 70.41 | 65.99 | 68.19 | 0.36 | 0.68 |
|  | Valid | 66.67 | 79.17 | 72.92 | 0.46 | 0.73 |
| NB | Train | 51.02 | 78.68 | 64.89 | 0.31 | 0.70 |
|  | Valid | 58.33 | 79.17 | 68.75 | 0.38 | 0.69 |
| J48 | Train | 63.27 | 61.42 | 62.34 | 0.25 | 0.63 |
|  | Valid | 66.67 | 54.17 | 60.42 | 0.21 | 0.66 |

Supplementary Table S14: List of experimentally verified AntiTbP (positive dataset) and their probability or prediction of being ABP or AMP by iAMP and DBAASP.

| S. No | AntiTbP Sequence | iAMP score | DBAASP result |
| --- | --- | --- | --- |
| 1 | AAARIRHEGVFLLIGNSCFSLPRNGPQLLLLAW | 0.032 | Non-AMP |
| 2 | AAPEPVARR | 0.06 | Non-AMP |
| 3 | AASAAIANR | 0.543 | Non-AMP |
| 4 | AGYLLGKINLKALAALAKKIL | 0.99 | AMP |
| 5 | ALADLPVTV | 0.166 | Non-AMP |
| 6 | AMASTEGNV | 0.149 | Non-AMP |
| 7 | AMEDLVRAY | 0.142 | Non-AMP |
| 8 | AMLGHAGDM | 0.616 | Non-AMP |
| 9 | ANNTRLWVY | 0.754 | Non-AMP |
| 10 | ANTMAMMAR | 0.644 | Non-AMP |
| 11 | APKGVQGPNG | 0.335 | Non-AMP |
| 12 | ATCDLLSGTGINHSACAAHCLLRGNRGGYCNGKAVCVCRN | 0.988 | AMP |
| 13 | ATLHLVLRLRGG | 0.039 | Non-AMP |
| 14 | ATYYGNGLYCNKQKHYTWVDWNKASREIGKITVNGWVQH | 0.969 | AMP |
| 15 | AVAGEKLWLLPHLLKMLLTPTP | 0.013 | Non-AMP |
| 16 | AVALGLASPADAAAGTMYGD | 0.15 | Non-AMP |
| 17 | AYQGVQQKW | 0.518 | AMP |
| 18 | CLLKKLLKK | 0.962 | AMP |
| 19 | CLLKKLLKKC | 0.95 | AMP |
| 20 | CNGKRVCVCR | 0.977 | Non-AMP |
| 21 | DAACAAHCLFR | 0.291 | Non-AMP |
| 22 | DEPDAERFEAAVEADHI | 0.085 | Non-AMP |
| 23 | DTHFPICIFCCGCCHRSKCGMCCKT | 0.997 | AMP |
| 24 | ECYRKSDIVTCEPWQKFCYREVTFFPNHPVYLSGCASECTETNSKWCCTTDKCNRARGG | 0.992 | Non-AMP |
| 25 | EFAGAGFVRAGAL | 0.117 | Non-AMP |
| 26 | ELAAIRHR | 0.194 | Non-AMP |
| 27 | ELNNALQNL | 0.364 | Non-AMP |
| 28 | ELNNALQNLART | 0.068 | Non-AMP |
| 29 | ESTYQGHHTPPVQKGLRYGIILFITSEVFFFAGFF | 0.034 | Non-AMP |
| 30 | EVEPSDTIENVKAKIQ | 0.003 | Non-AMP |
| 31 | FFIYVWRRR | 0.652 | Non-AMP |
| 32 | FFLLSLIPSAISAIKKI | 0.981 | Non-AMP |
| 33 | FIKWKFRWWKWRK | 0.839 | AMP |
| 34 | FIYAGSLSA | 0.231 | Non-AMP |
| 35 | FKCKKWQWKMKKLG | 0.842 | Non-AMP |
| 36 | FKCRRWQWRMKKLG | 0.71 | Non-AMP |
| 37 | FLTSELPQW | 0.067 | Non-AMP |
| 38 | FRCRRWQWRMRRLG | 0.725 | AMP |
| 39 | FVYGNGVTSILVQAQFLVNGQRRFFYTPDK | 0.156 | Non-AMP |
| 40 | FWGALAKGALKLIGPGSLFSSFSKKD | 0.981 | Non-AMP |
| 41 | FWGALAKGALKLIGSLFSSFSKKD | 0.984 | AMP |
| 42 | FWGALAKGALKLIPSLFSSFSKKD | 0.944 | AMP |
| 43 | FWGFLGKLAMKAVPSLIGGNKK | 0.995 | AMP |
| 44 | FWGFLGKLAMKAVPSLIGGNKSSSK | 0.983 | AMP |
| 45 | FWGLKGLKGPGKFSKKL | 0.989 | AMP |
| 46 | FWGLKGLKKFSKKL | 0.985 | AMP |
| 47 | GFGCPFNARRCHRHCRSIRRRAGYCAGRLRLTCTCVR | 0.951 | Non-AMP |
| 48 | GFGCPLNQGACHRHCRSIRRRGGYCSGIIKQTCY | 0.993 | Non-AMP |
| 49 | GIGAVLKVLTTGLPALISWIKRKRQQ | 0.5 | AMP |
| 50 | GIGKFLHSAKKFGKAFVGEIMNS | 0.994 | AMP |
| 51 | GIGKFLKSKGKFGKA | 0.999 | AMP |
| 52 | GKKKKSVQWCA | 0.97 | Non-AMP |
| 53 | GLLKFIKKLL | 0.999 | AMP |
| 54 | GLLKRIKTLL | 0.921 | AMP |
| 55 | GLLRKGGEKIGEKLKKIGQKIKNFFQKLVPQPEQ | 0.934 | AMP |
| 56 | GNNKPVYIPRPRPPHPRLV | 0.942 | Non-AMP |
| 57 | GNNRPVYIPQPRPPHPRI | 0.949 | AMP |
| 58 | GNNRPVYIPRPRPPHPRL | 0.969 | Non-AMP |
| 59 | GRRRRSVQWCA | 0.471 | AMP |
| 60 | GVENVSW | 0.722 | Non-AMP |
| 61 | GWGSFFKKAAHVGKHVGKAALTHYL | 0.987 | AMP |
| 62 | GWLKKIGKKIERVGQHTRDATIQGLGIAQQAANVAATARG | 0.919 | AMP |
| 63 | GYPWWDYRDLFGGHTFI | 0.161 | Non-AMP |
| 64 | GYPWWDYRDLFGGHTFISP | 0.073 | Non-AMP |
| 65 | HAMSSTHEA | 0.388 | Non-AMP |
| 66 | HQFRFRFRVRRK | 0.871 | AMP |
| 67 | IFSAIAGLLSNLL | 0.947 | Non-AMP |
| 68 | ILLKKLLKKI | 0.986 | AMP |
| 69 | ILPWKWRWWKWRR | 0.893 | Non-AMP |
| 70 | ILRWKWRWWRWRR | 0.871 | Non-AMP |
| 71 | ILSLRWRWKWWKK | 0.86 | AMP |
| 72 | IMYNYPAML | 0.626 | Non-AMP |
| 73 | INWLKLGKMVIDAL | 0.921 | AMP |
| 74 | IRMRIRVLL | 0.634 | AMP |
| 75 | IVLVRRWPK | 0.325 | AMP |
| 76 | IYAGSLSAL | 0.45 | Non-AMP |
| 77 | KEFKRIVKRIKKFLRKL | 0.99 | AMP |
| 78 | KEFKRIVKRIKKFLRKLV | 0.99 | AMP |
| 79 | KEFKRIVQRIKDFLRNLV | 0.8 | AMP |
| 80 | KFKWWRMLI | 0.76 | Non-AMP |
| 81 | KILRGVCKKIMRTFLRRISKDILTGKK | 0.99 | AMP |
| 82 | KIWWWWRKR | 0.74 | AMP |
| 83 | KKALAHALKKWLPALKKLAHALAKK | 0.94 | AMP |
| 84 | KKLALALAKKWLALAKKLALALAKK | 0.92 | AMP |
| 85 | KKLALALAKKWLPLAKKLALALAKK | 0.95 | AMP |
| 86 | KKLALHALKKWLHALKKLAHLALKK | 0.92 | AMP |
| 87 | KKLALLALKKWLLALKKLALLALKK | 0.61 | AMP |
| 88 | KKLALLALKKWLPALKKLALLALKK | 0.95 | AMP |
| 89 | KKLLFKLKFK | 0.95 | Non-AMP |
| 90 | KKVVFKFKFK | 0.76 | Non-AMP |
| 91 | KKVVFKVKFK | 0.81 | Non-AMP |
| 92 | KKVVPKVKFK | 0.83 | Non-AMP |
| 93 | KKVVVKVKFK | 0.76 | Non-AMP |
| 94 | KLLKFIKKLL | 0.99 | AMP |
| 95 | KLVANNTRL | 0.14 | Non-AMP |
| 96 | KMHATNH | 0.56 | Non-AMP |
| 97 | KMHATNHGGGS | 0.61 | Non-AMP |
| 98 | KPKGMTSSQWFKIQHMQPSPQASNSAMKNINKHTKRSKDLNTFLH | 0.14 | Non-AMP |
| 99 | KRKKRFKWW | 0.73 | AMP |
| 100 | KRLFKKLLFSLRKY | 0.92 | AMP |
| 101 | KRRWRIWLV | 0.74 | AMP |
| 102 | KRWWKWIRW | 0.77 | AMP |
| 103 | KRWWKWWRR | 0.78 | AMP |
| 104 | KRWWRKWWR | 0.79 | AMP |
| 105 | KRWWWWRFR | 0.8 | AMP |
| 106 | KTCENLADTY | 0.23 | AMP |
| 107 | KTLTGKTITLE | 0.71 | Non-AMP |
| 108 | KTYYGTNGVHCTKNSLWGKVRLKNMKYDQNTTYMGRLQDILLGWATGAFGKTH | 0.77 | Non-AMP |
| 109 | KWKLFKKIGAVLKVL | 1 | AMP |
| 110 | KWKLFKKIGIGAVLKVLTTGLPALIS | 0.99 | AMP |
| 111 | KWKSFIKKLTSAAKKVVTTAKPLISS | 0.95 | AMP |
| 112 | KWKSFLKTFKSAKKTVLHTALKAISS | 0.98 | AMP |
| 113 | KWKSFLKTFKSAKKTVLHTLLKAISS | 0.97 | AMP |
| 114 | KWKSFLKTFKSLKKTKLHTLLKLISS | 0.96 | AMP |
| 115 | KWKSFLKTFKSLKKTVLHTLLKAISS | 0.96 | AMP |
| 116 | KWKSFLKTFKSLKKTVLHTLLKLISS | 0.94 | AMP |
| 117 | KWKWWWRKI | 0.71 | AMP |
| 118 | KYIAADRKI | 0.83 | Non-AMP |
| 119 | KYIFPGGLL | 0.7 | Non-AMP |
| 120 | LKRRWKWWI | 0.85 | AMP |
| 121 | LLDEGKQSL | 0.19 | Non-AMP |
| 122 | LLGDFFRKSKEKIGKEFKRIVQRIKDFLRN | 0.96 | AMP |
| 123 | LLGDFFRKSKEKIGKEFKRIVQRIKDFLRNLVPRTES | 0.6 | AMP |
| 124 | LLIILRRRIRKQAHAHSK | 0.66 | AMP |
| 125 | LLKKLLKK | 0.95 | AMP |
| 126 | LLKKLLKKC | 0.966 | AMP |
| 127 | LLKKLLKKM | 0.928 | AMP |
| 128 | LPFFLLSLIPSAISAIKKI | 0.973 | AMP |
| 129 | LPQWLSANR | 0.017 | Non-AMP |
| 130 | LRFILWWKR | 0.809 | Non-AMP |
| 131 | LRRWIRIRW | 0.684 | AMP |
| 132 | LVRAYHAMS | 0.205 | AMP |
| 133 | LVYGDVIMR | 0.276 | Non-AMP |
| 134 | MLGHAGDMA | 0.616 | Non-AMP |
| 135 | MLLKKLLKK | 0.925 | AMP |
| 136 | MLLKKLLKKM | 0.814 | AMP |
| 137 | NDAACAAHCLFRGRSGG | 0.337 | Non-AMP |
| 138 | NEDTVTQAASRVCDKMKILRGVCKKIMRTFLRRISKD | 0.402 | AMP |
| 139 | NGKRVCVCR | 0.953 | Non-AMP |
| 140 | NVTSIHSLL | 0.313 | Non-AMP |
| 141 | NWRKLYRRK | 0.708 | AMP |
| 142 | PLLKKLLKKP | 0.92 | AMP |
| 143 | PRVIETKVHGREVTGLARNVSEENVDRLAKRWIK | 0.005 | AMP |
| 144 | PYNLRYRVL | 0.596 | Non-AMP |
| 145 | QIMYNYPAM | 0.611 | Non-AMP |
| 146 | QRSVSNAATRVCRTGRSRW | 0.136 | AMP |
| 147 | QTYKWETFL | 0.391 | Non-AMP |
| 148 | RAGLQFPVGRVHRLLRK | 0.534 | AMP |
| 149 | RFGEPGGRE | 0.137 | Non-AMP |
| 150 | RIKRWWWWR | 0.792 | AMP |
| 151 | RIRRWKFRW | 0.807 | AMP |
| 152 | RIWVIWRR | 0.732 | Non-AMP |
| 153 | RKDVY | 0.552 | Non-AMP |
| 154 | RKDVYRRRRRR | 0.769 | Non-AMP |
| 155 | RKFRWWVIR | 0.851 | AMP |
| 156 | RKSAKKIGKRAKR | 0.966 | AMP |
| 157 | RKSKEKIGKEFKRIVQRIKDFLRNLVPRTES | 0.423 | AMP |
| 158 | RKWKIKWYW | 0.754 | AMP |
| 159 | RLGRLVSLHTLG | 0.057 | Non-AMP |
| 160 | RLKRWWKFL | 0.815 | AMP |
| 161 | RLLKFIKKLL | 0.994 | AMP |
| 162 | RLRRIVVIRVFR | 0.878 | AMP |
| 163 | RLWRIVVIRVKR | 0.653 | AMP |
| 164 | RLWWKIWLK | 0.84 | AMP |
| 165 | RLWWWWRRK | 0.793 | Non-AMP |
| 166 | RMWELYLAY | 0.545 | Non-AMP |
| 167 | RPPQFTRAQWFAIQHISLMPPRCTIAMRAINNYRWRCKNQNTFLR | 0.438 | Non-AMP |
| 168 | RPPQFTRAQWFAIQHISLNPPRSTIAMRAINNYRWRSKNQNTFLR | 0.136 | Non-AMP |
| 169 | RQIKIWFQNRRMKWKK | 0.763 | AMP |
| 170 | RQRRVVIWW | 0.711 | AMP |
| 171 | RRRIKIRWY | 0.795 | AMP |
| 172 | RRRPRPPYLPRPRPPPFFPPRLPPRIPPGFPPRFPPRFP | 0.945 | AMP |
| 173 | RRRRRR | 0.656 | Non-AMP |
| 174 | RRRRRRRKDVY | 0.763 | Non-AMP |
| 175 | RRRWWKLMM | 0.658 | AMP |
| 176 | RRWKIVVIRWRR | 0.672 | AMP |
| 177 | RRWRIVVIRVRR | 0.791 | AMP |
| 178 | RRWRVIVKW | 0.737 | AMP |
| 179 | RRWWKWWWR | 0.685 | AMP |
| 180 | RRWWRWVVW | 0.745 | AMP |
| 181 | RRYHWRIYI | 0.684 | AMP |
| 182 | RSGGYCNGKRVCVCR | 0.993 | Non-AMP |
| 183 | RTISEAGQAM | 0.1 | Non-AMP |
| 184 | RTKKWIVWI | 0.745 | AMP |
| 185 | RWRRKWWWW | 0.678 | Non-AMP |
| 186 | RWRWWWRVY | 0.76 | Non-AMP |
| 187 | RWWIRIRWH | 0.761 | AMP |
| 188 | RWWRKIWKW | 0.768 | AMP |
| 189 | RWWRWRKWW | 0.694 | Non-AMP |
| 190 | RWYYGNGVGGVGGAAVCGLAGYVGEAKENIAGEVRKGWGMAGGFTHNKACKSFPGSGWASG | 0.968 | Non-AMP |
| 191 | SALHLVLRLRGG | 0.132 | Non-AMP |
| 192 | SARLAGIPY | 0.108 | Non-AMP |
| 193 | SEAYQGVQQ | 0.334 | Non-AMP |
| 194 | SEAYQGVQQK | 0.354 | Non-AMP |
| 195 | SEFAYGSFVRTVSLPV | 0.005 | Non-AMP |
| 196 | SEFAYGSFVRTVSLPVGADE | 0 | Non-AMP |
| 197 | SGSEAYQGVQQKWDA | 0.136 | Non-AMP |
| 198 | SIIIPTLNV | 0.444 | Non-AMP |
| 199 | STAHLVLRLRGG | 0.027 | Non-AMP |
| 200 | STLALVLRLRGG | 0.013 | Non-AMP |
| 201 | STLHAVLRLRGG | 0.026 | Non-AMP |
| 202 | STLHLALRLRGG | 0.032 | Non-AMP |
| 203 | STLHLVARLRGG | 0.026 | Non-AMP |
| 204 | STLHLVLALRGG | 0.041 | Non-AMP |
| 205 | STLHLVLRARGG | 0.027 | Non-AMP |
| 206 | STLHLVLRLAGG | 0.044 | AMP |
| 207 | STLHLVLRLRAG | 0.014 | Non-AMP |
| 208 | STLHLVLRLRGA | 0.014 | Non-AMP |
| 209 | STLHLVLRLRGG | 0.054 | Non-AMP |
| 210 | SVFPQQTTGQLAELQPQDRAGARASWMPMFQRRRRR | 0.155 | Non-AMP |
| 211 | TATELNNAL | 0.174 | Non-AMP |
| 212 | TGMAALEQYLGSGHAVIVSI | 0.084 | Non-AMP |
| 213 | TKPKGTKPKGTKPKGTKPKG | 0.912 | Non-AMP |
| 214 | TQRSVSNAATRVCRTGRSRWRDVCRNFMRRYQSRVTQG | 0.552 | AMP |
| 215 | TTKNYGNGVCNSVNWCQCGNVWASCNLATGCAAWLCKLA | 0.992 | Non-AMP |
| 216 | VCDKMKILRGVCKKIMRSFLRR | 0.949 | AMP |
| 217 | VCDKMKILRGVCKKIMRTFLRR | 0.94 | AMP |
| 218 | VCEHIHLIRGLCHHLMHSYIKR | 0.86 | AMP |
| 219 | VCRTGRSRWRDVCRNFMRRYQSR | 0.816 | AMP |
| 220 | VCRTGRSRWRDVNRNFMRRYQSR | 0.643 | AMP |
| 221 | VEPIPY | 0.428 | Non-AMP |
| 222 | VLAGSVDEL | 0.179 | Non-AMP |
| 223 | VPAESEAAHLRVRRGFGCPLNQGACHNHCRSIRRRGGYCSGIIKQTCTCYRN | 0.96 | Non-AMP |
| 224 | VQLRIRVAVIRA | 0.533 | Non-AMP |
| 225 | VQRWLIVWRIRK | 0.451 | Non-AMP |
| 226 | VRLIVAVRIWRR | 0.522 | Non-AMP |
| 227 | VRLRIRVRVIRK | 0.915 | Non-AMP |
| 228 | VSNAATRVCRTGRSRWRDVCRNFMRRYQSR | 0.402 | AMP |
| 229 | VTCDILSVEAKGVKL | 0.438 | Non-AMP |
| 230 | WFKMRWWGR | 0.792 | Non-AMP |
| 231 | WGAQLNAMK | 0.786 | Non-AMP |
| 232 | WKIVFWWRR | 0.874 | Non-AMP |
| 233 | WKWLKKWIK | 0.737 | AMP |
| 234 | WKWRVRVTI | 0.738 | Non-AMP |
| 235 | WLLKKLLKKW | 0.75 | AMP |
| 236 | WRKFWKYLK | 0.794 | AMP |
| 237 | WRSLGRTLLRLSHALKPLARRSGW | 0.022 | AMP |
| 238 | WYYQSGLSI | 0.327 | Non-AMP |
| 239 | YCNGKRVCVCR | 0.991 | Non-AMP |
| 240 | YGRKKRRQRRR | 0.878 | Non-AMP |
| 241 | YKFRWRIYI | 0.599 | Non-AMP |
| 242 | YKQCHKKGGKKGSG | 0.971 | AMP |
| 243 | YLLDGLRAQ | 0.051 | Non-AMP |
| 244 | YPHHFKHRHIPI | 0.785 | Non-AMP |
| 245 | YPHHFKHRHIPIGGGS | 0.907 | Non-AMP |
| 246 | YRLRVKWKW | 0.784 | Non-AMP |
